# M. Devergie on Baths, Etc.

**Published:** 1855-01

**Authors:** 


### BUFFALO MEDICAL JOURNAL

AND

### MONTHLY REVIEW.

VOL. 10. JANUARY, 1855. NO. 8.

### ORIGINAL COMMUNICATIONS.

ART. I. — *M. Devergie on Baths, etc.* Translated from the French, by Prof. FLINT.

The following is a translation of the Chapter on Baths, etc., contained in the "*Practical Treatise on Diseases of the Skin,*" by M. ALPH. DEVERGIE, one of the attending physicians at the St. Louis Hospital, Paris. The translation is made in accordance with a partial promise contained in my letter from Paris relating to St. Louis Hospital:

TRANSLATION.

It appears to us important to devote a chapter specially to baths, mineral waters, and sea-bathing, not only because they are the most potent agencies in the management of diseases of the skin, but because very little has been written upon vapor baths and *douches*, and since most practitioners are satisfied simply to prescribe the use of waters without being able to give their patients any precise directions, relinquishing to the physicians of each establishment the entire direction of the method of administration. Without absolutely condemning this course, I will say that the practitioner, acquainted with the condition of his patient, ought to furnish him with some special instructions, and it is with reference to this view of the subject that I present the following considerations:

*Aqueous Baths.*

An aqueous bath acts in three modes, viz: by the liquid of which it is composed, by the temperature of this liquid, and the duration of the bath. As regards the first mode, water is absorbed as well as principles which it may hold in solution. The fact of absorption is demonstrated by persons urinating six, seven or eight times during a bath of an hour, or an hour and a half, although the bladder was empty immediately before entering the bath. However, in this respect different persons differ greatly, the organization of the skin in some being such that absorption is very active, while there are some persons whose dry skin absorbs but little, as there are some who almost never perspire.

The absorption of substances held in solution in water is rendered evident by the use of medicated baths, for instance those in which corrosive sublimate is dissolved, by means of which a very satisfactory mercurial treatment may be employed, curing constitutional syphilis quite as well as when this remedy is administered internally. Alkaline baths, those of Vichy for example, furnish still more decisive proof of this statement, so that it may be laid down as a general rule that water medicated either naturally or artificially, as sulphurous mineral waters, or those which are alkaline, saline, or impregnated with iron, are not restricted in their effects to the action exerted on the skin as a sedative, astringent, excitant or tonic, but their influence extends over the whole economy in consequence of their transporting to the blood the remedial elements which they contain.

An analogous absorption obtains in plants, which sustains the foregoing statement. Let a plant of any kind be sprinkled with a saline solution added to water in proportions not to prove injurious to vegetation, and there will be found in the vegetable, and all its products, leaves, flowers, and fruit, the substance contained in the water with which it had been sprinkled. Thus one can obtain salted grapes by watering the earth in which the vine grows with salt and water. The sulphate of iron, so extolled for its influence on vegetation, acts upon vegetables, which I call chloratic, or of sickly growth, as it acts on man. Soluble poisons even are absorbed by vegetables.

Hence, every practitioner who orders a bath or a mineral water, should bear in mind these facts, and consider the composition of the water with reference to absorption.

An aqueous bath not only acts by reason of its composition, but still more, by means of its temperature; and the latter exerts an influence very potent upon the person who makes use of it. Between a very hot bath in which we immerse an infant threatened with cerebral congestion, and a cold bath in which an individual sweating freely is placed in hydropathic practice, there is an immense distance, the effects being altogether different.

A water bath which has an amount of heat agreeable to the person immersed, is an emollient and antiphlogistic bath; any increase of heat excites the circulation; and a considerable increase accelerates greatly the heart's action, inducing a certain amount of cerebral and pulmonary congestion, so that a person who dies in a bath of too great temperature, succumbs to congestion of the head and lungs.

A bath below the medium temperature above mentioned, is a sedative bath for persons in whom the circulation is very active. These persons warm their baths as is commonly said, and with truth.

A bath colder, becomes tonic, and an excitant provided that one remains in it a short time only. At a temperature still more reduced, it induces, like the hot bath, pulmonary and cerebral congestions, but by another mode of action, viz: suppressing the eccentric capillary circulation, and driving, so to speak, the blood within.

If cold baths are employed by a person in full perspiration, it is the perspiration provoked by sheets wet in cold water which exerts a sedative action, and by immersion for some seconds in cold water, a reäction follows which strengthens the patient. The instantaneous contact of cold water produces this effect; prolonged contact may prove fatal by repelling from the periphery the blood to the lungs and brain.

The temperature cannot but be fixed upon with some difficulty when the object is to direct a temperate bath, which I call an antiphlogistic bath; for it is always relative to the subject. A bath, in my opinion, is really antiphlogistic when it is agreeable to the person taking it.

The duration of a bath, as must be at once evident, has a great influence on its therapeutic action, but this influence is altogether relative. With reference to ordinary baths there are persons to whom an ordinary bath prolonged for half an hour, proves debilitating. In such cases a half-hour bath is equivalent to a bath of an hour for the generality of persons. Persons of a bilious or a bilio-sanguine temperament frequently take baths of two or three hours duration without being in the least degree enfeebled. A duration of a hot bath equal to that of a temperate bath, is much more debilitating. A cold river bath of fifteen or twenty minutes duration, is a tonic bath provided the person knows how to swim, but it suffices to remain in the water eight or ten minutes to obtain the same result if this be not the case.

Cold river baths prolonged for two or three hours, even with swimming, are essentially debilitating, and with persons of a lymphatico-sanguine temperament they predispose to rheumatism, if, indeed, they are not adequate to induce it.

All these statements undergo modifications when applied to baths artificially medicated, or when the water is derived from mineral sources. The mean duration of a mineral bath is from three-quarters of an hour to an hour, but in some cases this period may be prolonged in consequence of the nature of the water, and the custom of the locality, the latter determined by experience. Thus, as a general remark, a pool-bath (*de piscine*) is supported longer than one taken in a bathing-house; for example, in the pools of Louesche one can remain every day for three, five and seven consecutive hours.

Bathing in a running stream may always be more prolonged than bathing in cold water. Baths taken in running water have an action altogether special on the skin, which baths in still water do not have. What is this action? We cannot define it in precise terms. It is exerted by the friction of the water on the skin, or by the more rapid subtraction of caloric in multiplying the points of contact of the liquid. How it is we are not prepared to say, but the action is much more energetic. At the baths of Bigorre there is a pool in which the water rises almost in surges. This pool is constantly filled with bathers, and this preference is thus marked because baths of that kind are much more efficacious in relieving neuralgic affections for which they are employed. The water which rises there is of the same nature as in the grand establishment.

In conclusion, when the practitioner prescribes a bath he must indicate precisely the duration according to the kind of bath, and the state of the patient.

### *Liquid Douches.*

These are of two kinds: douches in which the liquid is applied in a single jet, and those in which it is applied by showering. In the former case a jet is made to flow from a spout, the size varying from one extremely small, to one of a diameter of eight or ten *centimètres.** Larger than this the *douche*

* It is troublesome to reduce the French measures to English with exactness. A *mètre* is 1.093633 yard, a *centimètre* is a hundredth, and a *millemètre* a thousandth of a *mètre.*

receives the name of "*flot*," as it is called in hydropathy. It is evident that when the mass of water is considerable, it is a stream rather than a *douche;* for the latter supposes a fall of water from a greater or less height, but preserving in its course the form of a small column; whilst the *flot* sometimes falls in a sheet from a great height, and sometimes it escapes in a column from a reservoir but little elevated, else it would be impossible to support the force of the blow. Generally the jet of a *douche* varies from a diameter of two or three *millemètres*, to one of a *centimètre* and a half.

*Douches* by showering are composed of a series of jets more or less fine, which escape simultaneously from one point.

Few *douches*, either of a single jet, or by showering, in establishments for the use of mineral waters, or in those of Paris, either in the hospitals, or in the city, fulfill the conditions which one ought to find in them, viz: a jet neither twisted nor divided, preserving the same diameter for the distance of a *mètre*, the surface representing the clearness of a mirror. This depends on the formation of the socket for the stop-cock, and the form given to the aperture through which the stream flows. One is surprised in looking at the water running from the casks of the water-carriers in Paris, at the purity of the stream, but on going into a bathing establishment, sockets for stop-cocks are rarely found that are not badly made, and far from having the perfection of the apertures in these water-casks. This may seem almost puerile, or, at least, dealing with the *minutiæ* of the administration of *douches*, but I desire to call attention to the importance of such points, in order that the medical inspectors of mineral-waters may be led to introduce improvements in the contrivances by which these waters are applied.

A liquid *douche* has two quite different kinds of action, viz:

1st. It exerts an influence determined by the nature of the water. This mode we will not stop to consider.

2d. Its action is altogether different according as it is by jet, or showering.

It is necessary to have employed these therapeutical agents in order to understand their importance. The *douche* by jet produces on the skin the sensation of having been struck with a hammer. In this point of view it is calculated to blunt the sensibility. It is thus suited to neuralgies and affections in which there is a venous sur-excitation of the skin, like that occasioned by phlebitis. The *douche* by showering, on the contrary, stimulates the skin, causing a stinging, pricking sensation, which, when it is powerful, is like the points of needles exciting the skin in a painful degree. This kind of *douche* is, therefore, well suited to paralytic cases. This distinction, not heretofore made, as regards the effects of *douches*, is of great importance, and one can readily conceive the necessity of having properly constructed means of application, for if the small jets are confused, crossing each other, breaking, and reüniting in their course, the effect is not the same, the *douche* is useless. It is generally imagined that the vessel for showering must be of large size to furnish many jets. This is an error. A small surface, a diameter of two or three *centimètres*, suffices for all purposes.

Assuming the appendages to be well constructed, it is necessary that a *douche* have a great height, and that the reservoir be of a given form. A *douche* from a reservoir six *mètres* in depth, has a feeble power; but in exceeding this distance a great force is at once attained, one of ten *mètres* producing a certain action. It is of importance that the reservoir have its capacity in depth, not in width.

Moreover, with provisions for the most powerful *douches*, the feeblest may be administered. For this it suffices to turn the stop-cock one quarter or a half, so that there is a great advantage in having a reservoir of great depth. The practitioner has so often to prescribe the use of these measures that I feel bound to enter into such details. There are, besides, several modes in which they are to be employed. Either they may be given alone, without bathing, that is to say, the patient, being naked, is seated in a chair, or extended in a bathing-tub, and the water allowed to flow, after which he is immediately dressed and made to walk rather briskly, if he be able, in order to sustain the excitant action of the *douche;* or, on the contrary, if we have to do with a nervous, impressible subject in whom we fear to produce too much excitation, it is necessary that the *douche* be received in a bathing-tub, and immediately afterward a simple bath be taken, which may be continued a half-hour, in order to diminish the sar-excitation caused by the *douche.* In reality, a *douche* always gives rise to some disturbance of the nervous system which a bath immediately calms.

A *douche* may be local or general. When it is local, it is highly important sometimes to guard other parts of the body against the water, especially in winter. It is often useful, in applying a cold *douche* to the head, to place, at the same time, the patient in a very warm half-bath, in order to invite the blood to the extremities. In such cases the patient is efficiently protected by a small cloak of India-rubber cloth. In bathing establishments there are divers means for preserving certain parts of the body from the water of the *douche,* such as wooden fans or screens of some kind for the face when the *douche* is applied to the shoulders. A mode which we often adopt in cases of *acne rosacea* when the *douche* is directed on the face, is to apply a covering of caoutchouc adapted to the mouth, connected with a tube sufficiently long and wide, through which the patient respires without difficulty. This covering is fixed by two strings passing around the head above the ears.

[To be continued.]

